# White matter changes measured by multi-component MR Fingerprinting in multiple sclerosis

**DOI:** 10.1016/j.nicl.2023.103528

**Published:** 2023-10-11

**Authors:** Martijn A. Nagtegaal, Ingo Hermann, Sebastian Weingärtner, Eloy Martinez-Heras, Elisabeth Solana, Sara Llufriu, Achim Gass, Dirk H.J. Poot, Matthias J.P. van Osch, Frans M. Vos, Jeroen de Bresser

**Affiliations:** a Department of Imaging Physics, Delft University of Technology, Delft, the Netherlands; b C.J. Gorter MRI Center, Department of Radiology, Leiden University Medical Center, Leiden, the Netherlands; c Computer Assisted Clinical Medicine, Medical Faculty Mannheim, Heidelberg University, Mannheim, Germany; d Neuroimmunology and Multiple Sclerosis Unit and Laboratory of Advanced Imaging in Neuroimmunological Diseases (ImaginEM). Hospital Clinic Barcelona, Institut d’Investigacions Biomediques August Pi i Sunyer (IDIBAPS) and Universitat de Barcelona, Barcelona, Spain; e Department of Neurology, Medical Faculty Mannheim, Heidelberg University, Mannheim, Germany; f Department of Radiology and Nuclear Medicine, Erasmus MC, Rotterdam, the Netherlands; g Department of Radiology, Leiden University Medical Center, Leiden, the Netherlands

**Keywords:** Multiple Sclerosis, White matter damage, MR Fingerprinting, Quantitative MRI, Multi-component analysis

## Abstract

*T*_2_-hyperintense lesions are the key imaging marker of multiple sclerosis (MS). Previous studies have shown that the white matter surrounding such lesions is often also affected by MS. Our aim was to develop a new method to visualize and quantify the extent of white matter tissue changes in MS based on relaxometry properties.

We applied a fast, multi-parametric quantitative MRI approach and used a multi-component MR Fingerprinting (MC-MRF) analysis. We assessed the differences in the MRF component representing prolongedrelaxation time between patients with MS and controls and studied the relation between this component’s volume and structural white matter damage identified on FLAIR MRI scans in patients with MS.

A total of 48 MS patients at two different sites and 12 healthy controls were scanned with FLAIR and MRF-EPI MRI scans. MRF scans were analyzed with a joint-sparsity multi-component analysis to obtain magnetization fraction maps of different components, representing tissues such as myelin water, white matter, gray matter and cerebrospinal fluid. In the MS patients, an additional component was identified with increased transverse relaxation times compared to the white matter, likely representing changes in free water content. Patients with MS had a higher volume of the long- component in the white matter of the brain compared to healthy controls (B (95%-CI) = 0.004 (0.0006–0.008), p = 0.02). Furthermore, this MRF component had a moderate correlation (correlation coefficient R 0.47) with visible structural white matter changes on the FLAIR scans. Also, the component was found to be more extensive compared to structural white matter changes in 73% of MS patients.

In conclusion, our MRF acquisition and analysis captured white matter tissue changes in MS patients compared to controls. In patients these tissue changes were more extensive compared to visually detectable white matter changes on FLAIR scans. Our method provides a novel way to quantify the extent of white matter changes in MS patients, which is underestimated using only conventional clinical MRI scans.

## Highlights

• Brain tissue changes in MS is more extensive than only (MRI) visually detectable white matter changes.
• We propose to use multi-component MR Fingerprinting to quantify more subtle brain changes.
• A component with relatively long is more present in patients with MS than in control subjects.
• The MRF-component showed more extensive tissue changes than conventional MRI sequences.

## 1 Introduction

Multiple Sclerosis (MS) is a demyelinating disease of the central nervous system. A key imaging marker of MS are *T*_2_-hyperintense lesions, that appear mainly in the white matter of the brain and are particularly visible in *T*_2_-weighted and *T*_2_-Fluid-attenuated inversion recovery (FLAIR) MR images (Filippi et al., 2016, Wardlaw et al., 2015). So-called’dirty appearing white matter’ (DAWM) is another structural abnormality of MS that can often be distinguished in the white matter surrounding the *T*_2_-hyperintense lesions. DAWM is defined as areas of white matter that are mildly hyperintense on *T*_2_ compared to the (surrounding) normal appearing white matter (Cairns et al., 2022, Laule et al., 2013). However, these structural brain abnormalities by themselves are not able to fully capture the extent of white matter changes caused by MS (Filippi et al., 2019, Moll et al., 2011).

Different MR methods have been previously used to study white matter changes in MS to show microstructural changes in normal appearing white matter (i.e. no *T*_2_-hyperintense lesions and/or DAWM). Within visually normal appearing white matter previous studies showed decreases in magnetization transfer, *R*_1_(=1*/T*_1_), and total sodium concentrations in MS patients compared to healthy controls, indicating that there are more subtle changes in the white matter in MS than visible on structural MRI scans (Lommers et al., 2019, Weber et al., 2021). MRI diffusion based metrics have also demonstrated to be sensitive to changes in the normal appearing white matter in MS patients (Raja et al., 2019, Werring et al., 1999, De Santis et al., 2019). In earlier MRI diffusion studies (Filippi et al., 2001, Werring et al., 1999) it was observed that mean diffusivity was increased and the averaged fractional anisotropy was decreased in the normal appearing white matter in MS patients compared to healthy controls. In later imaging and histopathologic analyses, DAWM regions were identified in the previously perceived visually normal appearing white matter. These DAWM regions showed decreased fractional anisotropy on MRI and on histopathological analyses showed a decreased myelin density, extensive axonal loss, and chronic fibrillary gliosis (Seewann et al. 2009). In more recent studies, more advanced diffusion measures such as restricted signal fraction showed to be sensitive to microstructural white matter changes in early MS patients (De Santis et al. 2019).

An important drawback of conventional diffusion imaging based MRI measures is the inability to isolate the signal contribution from different tissue compartments, thereby reducing the specificity for tissue subtypes and associated injuries or microstructural changes (Lakhani et al. 2020). The more recent multi-compartment diffusion MRI models provide increased specificity, but also require long scan times (more than 15 min), show increased variation in parameter estimates or require high gradient hardware (Lakhani et al. 2020). Limitations of these previously used methods to study changes in the normal appearing white matter in MS is that the exact extent and severity of the white matter burden is difficult to capture quantitatively and visually at the same time.

Previous studies have also used advanced quantitative relaxometry MRI methods to assess the underlying microstructural changes that are present in *T*_2_-hyperintense lesions and DAWM. Such studies found that parameters including the *T*_1_*,T*_2_*,T*_1_*_ρ_* decay constants and water content, all reflected changes within *T*_2_-hyperintense lesions and DAWM compared to the normal appearing white matter (Cairns et al. 2022). However, these approaches require thresholds to differentiate between normal and abnormal tissue and are mostly hampered by lengthy acquisition times. To reduce acquisition times in quantitative MRI, Magnetic Resonance Fingerprinting (MRF) was proposed as an MR imaging framework. MRF combines transient state acquisitions, efficient k-space sampling trajectories and signal simulations to encode multiple MR parameters in a single acquisition (Ma et al., 2013). MRF-EPI (Rieger et al., 2018, Rieger et al., 2017) uses gradient echoes formed by flip angles with varying magnitude, varying timings and extra inversion pulses to create such a transient state signal that varies over time, encoding $T_{1},T_{2}^{*}$ and $M_{0}$. A slice-by-slice EPI readout is used for efficient k-space encoding and signals are simulated per slice.

In standard MRF methods the signal from every voxel in the image is matched to a signal dictionary calculated from specific combinations of relaxation times and possibly other parameters. However, such a single component model effectively characterizes every voxel by a single combination of relaxation times, under the assumption that the complete voxel is one homogenous tissue. This implies a drastic simplification of the complex structure of biological tissue as it does not take multi-component effects and partial volume in voxels into account .(Whittall et al., 1999)

To include these microstructural and multi-component effects, the measured MRF signal can be modeled as a linear combination of simulated signals, allowing for Multi component (MC)-MRF estimation, in which several components, with their individual relaxation times and magnetization fractions, together represent the signal in each voxel (Deshmane et al., 2019, McGivney et al., 2018, Tang et al., 2018). This method is similar as used for myelin water fraction imaging from multi-echo spin-echo data (Whittall et al., 1997), but the sequence is sensitive to both $T_{1}$ and $T_{2}^{*}$ instead of $T_{2}.$ MC-MRF allows for a more heterogenous description of every voxel, but makes accurate estimations more difficult due to the increased number of solutions. To aid solving this inverse problem, a joint sparsity regularization using the SPIJN algorithm was proposed that limits the number of components (tissues) used across the brain (Nagtegaal et al. 2020). Essentially for every voxel and across voxels the method looks for a small number of tissues identified by their relaxation times and scaled by a magnetization fraction that can explain the measured signal. For example, in healthy individuals MC-MRF has led to the identification of myelin water components that have relatively short relaxation times (Cencini et al., 2019, Cui et al., 2021, Nagtegaal et al., 2023).

We hypothesized that novel quantitative MC-MRF methods are able to visualize and quantify the extent of white matter changes caused by MS, not only in visible *T*_2_-hyperintense lesions, but also in the normal appearing white matter. Furthermore, the relatively short scan times make MC-MRF clinically more interesting compared to other advanced quantitative MR imaging methods that are sensitive to microstructural changes. To investigate our hypothesis we performed MC-MRF to visualize and quantify the extent of white matter tissue changes in MS. We evaluated our MC-MRF approach by assessing differences between patients with MS and controls and studied the relationship between MRF features and structural white matter changes visible on FLAIR MRI images of patients with MS.

## 2 Methods

### 2.1 Dataset

Data was acquired at 2 sites, on 3 T MRI scanners (Magnetom Skyra and Magnetom Prisma, Siemens Healthineers, Erlangen, Germany). At the first site (Medical Faculty Mannheim) 12 healthy volunteers and 18 patients with MS were scanned and at the second site (Hospital clinic Barcelona) 30 patients with MS were scanned. Previously, these data were used to analyze relaxation times in *T*_2_-hyperintense lesions as obtained from single component MRF approach (Hermann et al. 2021). This bicenter study was approved by the local institutional review board at both sites (2019–711 N, HCB2012/7965), and written, informed consent was obtained from all participants prior to scanning.

An MRF-EPI sequence (Rieger et al., 2018, Rieger et al., 2017) was applied with in-plane spatial resolution of 1 mm × 1 mm, slice thickness of 2 mm, bandwidth 998 Hz/px, GRAPPA factor 3, partial Fourier 5/8, fat suppression, variable flip angle (34^◦^−86^◦^), TE (21–81*.*5 ms), TR (3530–6570 ms) and approximately 3 global inversion pulses per minute, resulting in varying inversion times during the acquisition. At site 2 simultaneous multislice imaging was used with an acceleration factor of 3. The acquisition time for site 1 was 4 min and 23 s and 1 min and 52 s at site 2, at both sites covering 60 slices. *T*_2_-FLAIR and *T*_2_-weighted images were acquired using the same spatial resolution.

### 2.2 MRF reconstruction and image processing

**Fig. 1:**
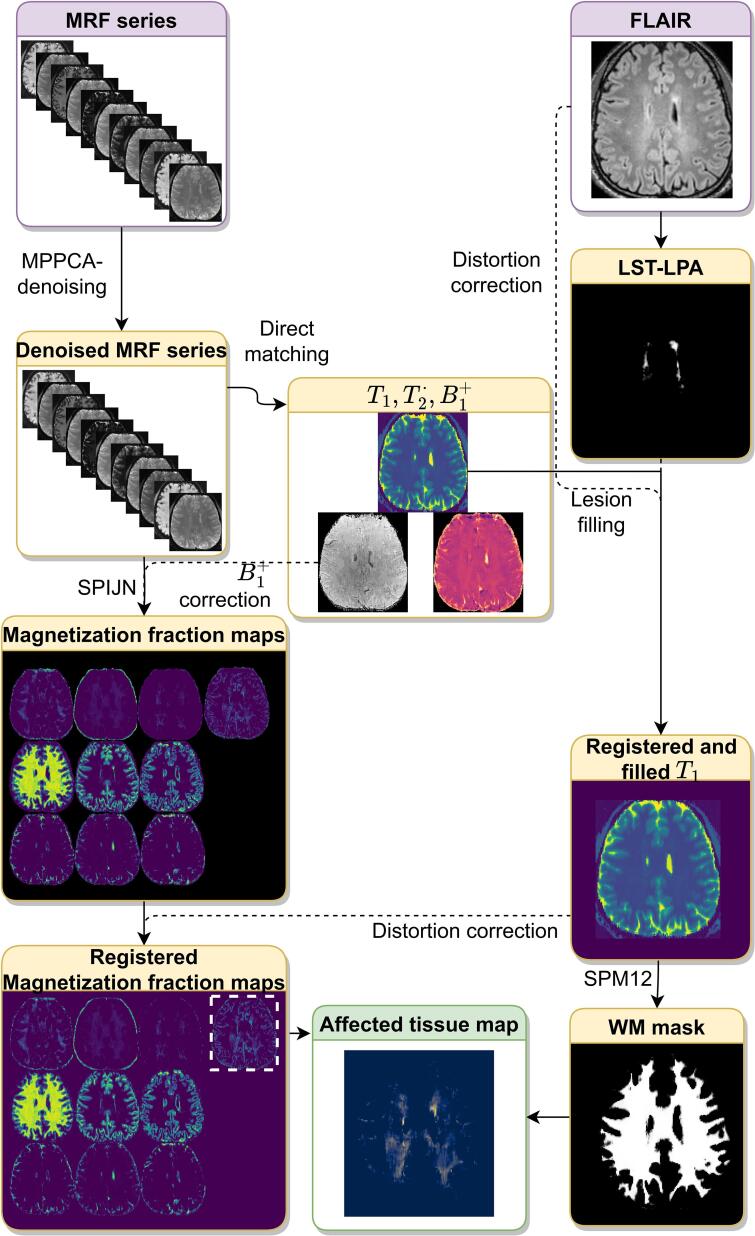
Flow diagram visualizing the proposed image processing steps. Marchenko-Pastur Principle Component Analysis (MP-PCA) denoising (Veraart et al. 2016) was used to denoise the MRF images. Lesion Segmentation Toolbox version 2.0.15 (Schmidt 2017) from which the Lesion Prediction Algorithm (LST-LPA) was used to obtain a lesion probability map for lesion filling. SPM12 (Ashburner and Friston 2005; SPM - Statistical Parametric Mapping 2019) was used to obtain white matter (WM) masks. The Sparsity promoting iterative joint NNLS (SPIJN) algorithm (Nagtegaal et al. 2020) was used to obtain Multi-component MRF magnetization fraction maps.

The further image processing steps described in this section are visualized in the flow diagram in Fig. 1.

For the development of the method as described in the following sections, a set of five subjects (3 site 1 and 2 site 2) was randomly selected to fine-tune parameter values based on visual inspection. After processing all processed images were visually checked for obvious errors (which were not found).

### 2.3 MRF processing

To analyze MRF data, a first required step is to simulate MRF signals for the used sequence, which form a dictionary used for extracting information in subsequent steps. MRF signal dictionaries were generated per slice using MATLAB (The MathWorks; Natick, MA, USA) consisting of 131,580 entries with *T*_1_ (30–4000 ms) in 5% steps, $T_{2}^{*}$ (5–3000 ms) in 5% steps, and flip angle efficiency (0.65–1.35) in steps of 0.05, excluding $T_{1}<T_{2}^{*}$. These ranges were chosen such that the total range of expected relaxation times was covered (Hermann et al. 2021).

Magnitude data of the MRF acquisition was denoised using Marchenko-Pastur Principle Component Analysis (MP-PCA) denoising (Veraart et al. 2016). Denoising was performed per slice and across timeframes in square patches of 7 voxels radius. Regular, single-component matching was performed on the denoised data to obtain and effective maps.

Subsequently, a joint sparsity, multi-component analysis was performed in a brain mask using the Sparsity Promoting Iterative Joint Non-negative least square (SPIJN) algorithm (Nagtegaal et al. 2020), in which the previously estimated map was used as fixed parameter. A normalized regularization *λ* = 11 (see Nagtegaal et al. 2020) was used for the MC-MRF analysis. In a pre-processing step, we obtained a brain mask by thresholding the *M*_0_ map (relative *M*_0_ > 5%) and identifying the largest connected region, namely the region containing the brain. This threshold (and also the ones used later) as well as regularization parameters were chosen based on 5 randomly selected datasets.

To correct for susceptibility induced geometric distortion, the MRF-EPI magnitude data was rigidly registered to the *T*_2_-weighted data followed by a restricted, nonlinear registration along the phase-encode direction of the magnitude data to the *T*_2_-weighted data using ANTs (Klein et al., 2009, Avants et al., 2009).

### 2.4 Conventional segmentation

*T*_2_-hyperintense lesion segmentations were obtained using the lesion prediction algorithm from the Lesion Segmentation Toolbox version 2.0.15 (LPA-LST) (Schmidt 2017). Masks were obtained by applying a threshold of 0.5. Lesion filling was performed on the *T*_1_ maps using the lesion segmentation toolbox. White matter, gray matter and cerebrospinal fluid segmentations were obtained from the lesion-filled *T*_1_ maps using Statistical Parametric Mapping version 12 (SPM12) (Ashburner and Friston, 2005, SPM – Statistical Parametric Mapping, 2019).

Distortion corrected *T*_1_ maps were registered to the MNI152 template and corresponding atlas (ICBM 2009a Nonlinear Symmetric 1 mm × 1 mm × 1 mm template) (Fonov et al., 2011, Collins et al.,, Fonov et al., 2009) to obtain segmentations of the frontal, occipital, parietal and temporal lobes.

### 2.5 MRF postprocessing

The obtained magnetization fraction maps were compared to structural FLAIR images and relaxation times were compared to literature values for related tissues (Bojorquez et al., 2017, Nunez-Gonzalez et al., 2022). A priori, myelin water, gray matter, white matter and cerebrospinal fluid components were expected and potentially components related to lesions. When multiple clusters were identified close to each other, k-means clustering of the log-scaled values was performed and mean and standard deviation of the relaxation times per cluster were obtained.

As further described in Section 3.2, visual inspection revealed components with prolonged relaxation times compared to white matter and gray matter. Therefore, these components were further analyzed and for subjects in whom multiple component with longer were identified, all MC-MRFcomponents with relaxation times of 500 ms *< T*_1_ *<* 2500 ms and 2500 ms were combined into a single magnetization fraction map.

Our aim was to study white matter changes in MS, therefore further analysis focused on these long- MRF-component maps in the white matter. Non-white matter regions in the long- MRF-component maps were masked based on the SPM12 white matter segmentation. Volumes of the long- MRFcomponent maps were calculated for the different white matter regions, per hemisphere and for the complete white matter. Volumes were normalized between 0 and 1 with respect to the total white matter volume of the considered region. A threshold of 15% was set for regions that showed a strong correspondence to $T_{2}$-hyperintensities.

### 2.6 Differences in MC-MRF between patients with MS and controls

#### 2.6.1 Statistical analysis

All statistical tests were performed using the Statsmodels and Pingouin packages (0.5.2) (Vallat, 2018) in Python 3.8. p-values less than 0.05 were considered statistically significant.

Generalized linear regression analyses correcting for age and sex were performed to assess the difference in estimated normalized long- MRF-component volumes between patients with MS and controls. These analyses were performed for the total white matter volume, as well as for the white matter volume per brain lobe (frontal, occipital, parietal and temporal lobes, both left and right). Subsequently, the difference between patients with MS and controls in the volumes was assessed excluding the *T*_2_-hyperintense lesions. For these analyses, the obtained LPA-LST lesion probability maps (thresholded at 50%) were used to mask *T*_2_-hyperintense lesions from the long-MRF-component map and normalized volumes were calculated for the different white matter regions. Estimated volumes from the MC-MRF and LPA-LST were compared using a paired *t*-test to assess differences in affected white matter areas. As secondary analyses, linear regression analyses were performed to test for relations between estimated normalized volumes and EDSS, disease duration, sex and age. Additional sensitivity analyses comparing patients and controls as described above were performed with an age-restricted group and only considering Site A. This age restricted group was formed by removing patients older than 33 years, resulting in two groups of around the same mean age.

### 2.7 Relation between MC-MRF and structural white matter changes on FLAIR MRI in patients with MS

#### 2.7.1 Visual scoring

The *T*_2_-weighted and *T*_2_-FLAIR scans white matter regions were evaluated by an expert neuroradiologist (JB) on a 0–5 scale reflecting the amount of structural white matter changes; this expert assessed the combined DAWM and *T*_2_-hyperintense lesions and DAWM by itself. DAWM was defined as an uniform area of subtle signal intensity increase on *T*_2_-FLAIR compared to the signal intensity of the normal appearing white matter (Seewann et al. 2009). The following visual volume scores were applied:

1. No structural changes
2. 0% to 10% abnormal white matter of total WM
3. 10% to 25% abnormal white matter of total WM
4. 25% to 50% abnormal white matter of total WM
4. 50% to 75% abnormal white matter of total WM
5. 75% or more abnormal white matter of total WM

This scoring was performed per white matter region (frontal, parietal, occipital and temporal), and per hemisphere, resulting in a maximum score of 40 points. This non-linear score was used to increase sensitivity to small areas of affected tissue. Scores were summed to provide scores for the left and right hemisphere and for the complete brain.

#### 2.7.2 Visual similarity scoring

A similarity scoring was performed comparing the long- MRF-component maps to the extent of structural white matter changes on the FLAIR images (i.e. *T*_2_-hyperintense lesions and DAWM). The following scores were applied:

1. Less extensive involvement in the MC-MRF compared to FLAIR.
2. Similar involvement.
3. More extensive involvement in the MC-MRF compared to FLAIR.

For each white matter region a score was obtained for all structural white matter changes and another score for only *T*_2_-hyperintense lesions.

### 2.8 Statistical analysis

A Spearman pairwise correlation test was performed to test for correlations between estimated normalized volumes and the visual volume scoring within the patient group, while correcting for the two different sites. Tests were performed for the whole white matter and also per white matter region for each hemisphere. From the tests correlation coefficients, p-values and 95% confidence intervals were obtained.

## 3 Results

### 3.1 Dataset

At the first site 12 healthy controls (9 male, 3 female, mean age 26 years (range 22–30 years)) and 18 patients (7 male, 11 female, 39 years (23–73 years)) with MS were scanned. At the second site 30 patients (10 male, 42 years (26–62 years)) with MS were scanned. Table 1In Table 1 the characteristics of the scanned participants are summarized.

**Table 1:** Demographic and clinical characteristics of the scanned MS patients and healthy controls summarized as age, disease duration, the Expanded Disability Status Scale (EDSS) and disease type. Data are shown as n, mean ± SD or median (interquartile range). Normally distributed values (Anderson Normality test) are reported as mean and standard deviation (±), others as median and interquartile range. Note that age and sex between patients and healthy controls differs significantly. n.a.: not applicable, RRMS: relapsing remitting MS, PPMS: primary progressive MS, SPMS: secondary progressive MS, CIS: clinically isolated syndrome.

	**MS Patients (both sites)**	**MS Patients (Site A)**	**MS Patients (Site B)**	**Healthy controls (Site A)**
Total number	48	18	30	12
Female	31	11	20	3
Male	17	7	10	9
Age (years)	39 (32–46)	34 (29–44)	41 (36–48)	25 (23–29)
Disease duration (years)	5.2 (2.8–13.8)	5.5 (3.0–12.3)	5.2 (2.2–14.1)	n.a.
EDSS	2 (1–2.5)	1.5 (1.0–2.1)	2.0 (1.0–3.3)	n.a.
RRMS	41	17	24	n.a.
PPMS	0	0	0	n.a.
SPMS	5	0	5	n.a.
CIS	1	1	1	n.a.
Visual scoring of white matter changes	10.8 ± 4.4	9.7 ± 3.5	11.4 ± 4.8	7 ± 4.3

### 3.2 MC-MRF analysis

**Fig. 2:**
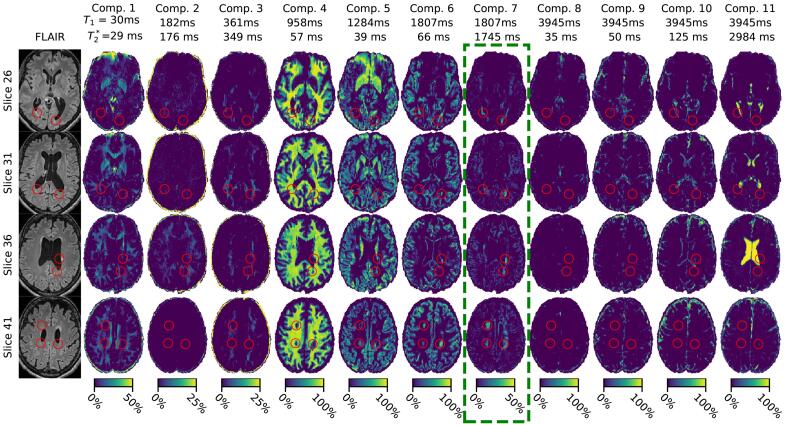
FLAIR images and obtained MC-MRF component maps a for a selection of slices of a representative MS patient. Estimated relaxation times are shown above each column. Note that color ranges differ per component for visualization purposes. Components are ordered by *T*_1_ relaxation time. Shown images are registered to correct for susceptibility distortion. The green box indicates the component of interest. The red circles indicate white matter damage as visible in component 7. Component 2 shows a sharp increase in signal in slice 36 compared to the other slices. This is most likely caused by the different timing of RF-pulses per slice used in the MRF-EPI sequence. (For interpretation of the references to color in this figure legend, the reader is referred to the web version of this article.)

**Fig. 3:**
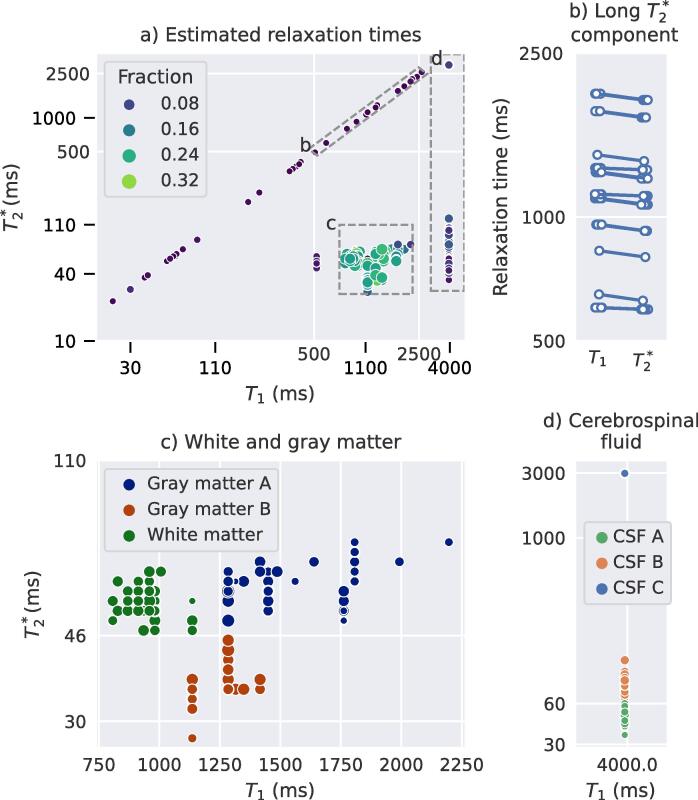
Estimated relaxation times from the MC-MRF analysis of all subjects. The size and color of the dots reflect the fraction of the component of the total volume a) Overview of all matched relaxation times; dots out of the 3 boxes mainly concerned myelin water like components; b) distribution of the components with long $T_{2}^{*}$ relaxation times, selected as 500 ms *< T*_1_ *<* 2500 ms and $500\text{ms}<T_{2}^{*}<$ 2500 ms; c) close up and clustering outcome for gray and white matter components and d) close up of cerebrospinal fluid related components.

Examples of the obtained FLAIR images and estimated relaxation times and magnetization fraction maps from the MC-MRF are shown in Fig. 2 for a single MS patient. In Fig. 2 it can be observed that components 1 to 3 contain short relaxation times (*T*_1_ *<* 500 ms) and mainly present white matter and deep gray matter regions. Such components might be related to myelin water given their visual appearance and short $T_{1}$ relaxation times. Component 4 is the main signal component in white matter regions. This component and the other component (groups) were estimated in all participants, as shown in Fig. 3; the means (and standard deviations) of the relaxivities were *T*_1_ = 970 ± 79 ms and 2 ms. Components 5 (having reduced value) and 6 as shown in Fig. 2 relate to cortical gray matter and deep gray matter regions. Over all participants component 5 had 46 ms; on average *T*_1_ = 1197 ± 81 ms and 3 ms. The other gray matter component (6) showed more variation in estimated relaxation times: *T*_1_ = 1494 ± 182 ms and 2 ms. Component 7 as shown in Fig. 2 corresponds to increased signal of the *T*_2_-hyperintense lesions as well as in other white matter areas on the FLAIR scan and is the component of our interest with long. Components 8 to 11 all have the maximum *T*_1_ relaxation time of around 4000 ms and correspond to cerebrospinal fluid regions. In most cases (89%) 3 components were identified with9ms, 82*.*9 ± 11*.*5 ms and 2984 ± 0 ms of which the last component always had the maximum value in our dictionary, in Fig. 3 these components were referred to as cerebrospinal fluid A, B and C respectively.

One or two components with 500 ms *< T*_1_ *<* 2500 ms, were observed in all participants with varying total volume and relaxation times (mean *T*_1_ = 1489 ± 667 ms and mean). The individual *T*_1_ and times were very close to each other as can also be observed in Fig. 3a. In the healthy subjects this component was limited in the white matter, whereas in patients this component corresponded to structural white matter changes as readily visible in Fig. 2.

**Fig. 4:**
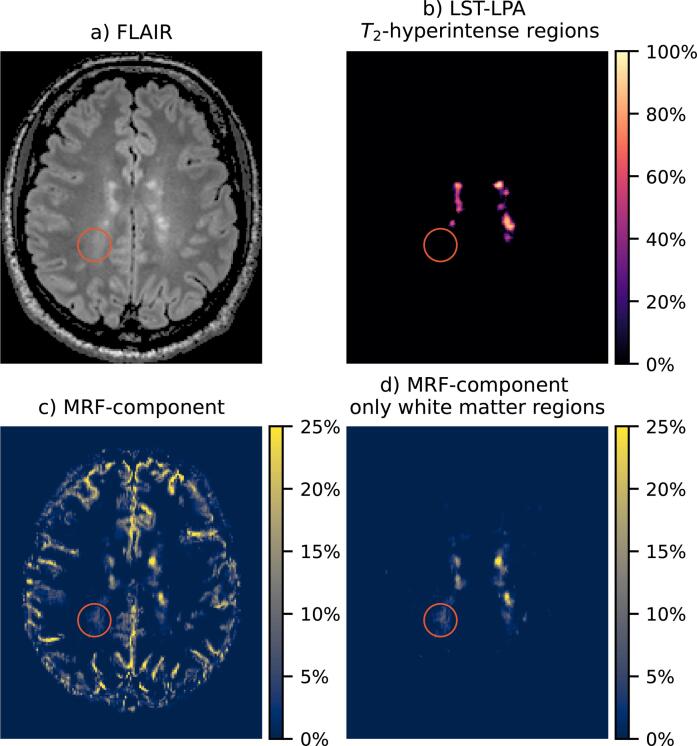
Results from a single slice of an MS patient. A region with DAWM is indicated by the red circle. a) FLAIR scan, b) estimated lesion probability map from the FLAIR scan, c) MRF-component with long before masking and d) after masking non-white matter regions. (For interpretation of the references to color in this figure legend, the reader is referred to the web version of this article.)

**Fig. 5:**
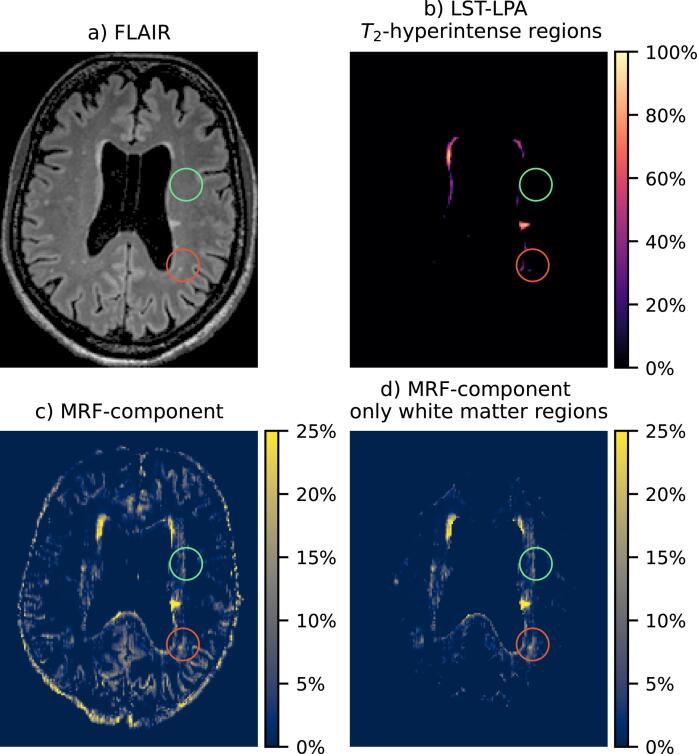
Results from a single slice of a second MS patient. A region with *T*_2_-hyperintense regions on the FLAIR scan is indicated with a red circle; a region reflecting white matter tissue changes visible only on the MRF map is indicated with a green circle. a) FLAIR scan, b) estimated lesion probability map from the FLAIR scan, c) MRF-component with long $T_{2}^{*}$ before masking and d) after masking non-white matter regions. (For interpretation of the references to color in this figure legend, the reader is referred to the web version of this article.)

Fig. 4 and Fig. 5 show detailed examples of the long- MRF-component next to the obtained FLAIR image and LST-LPA map. Fig. 4 shows a case in which the magnetization fraction maps show large similarity to lesions and DAWM in the FLAIR images, while Fig. 5 shows a representative case of extra signal compared to the FLAIR scan, c.f. the performed similarity scoring. In several cases the MRF component decreases gradually from the ventricles.

### 3.3 Differences in MC-MRF between patients with MS and controls

**Fig. 6:**
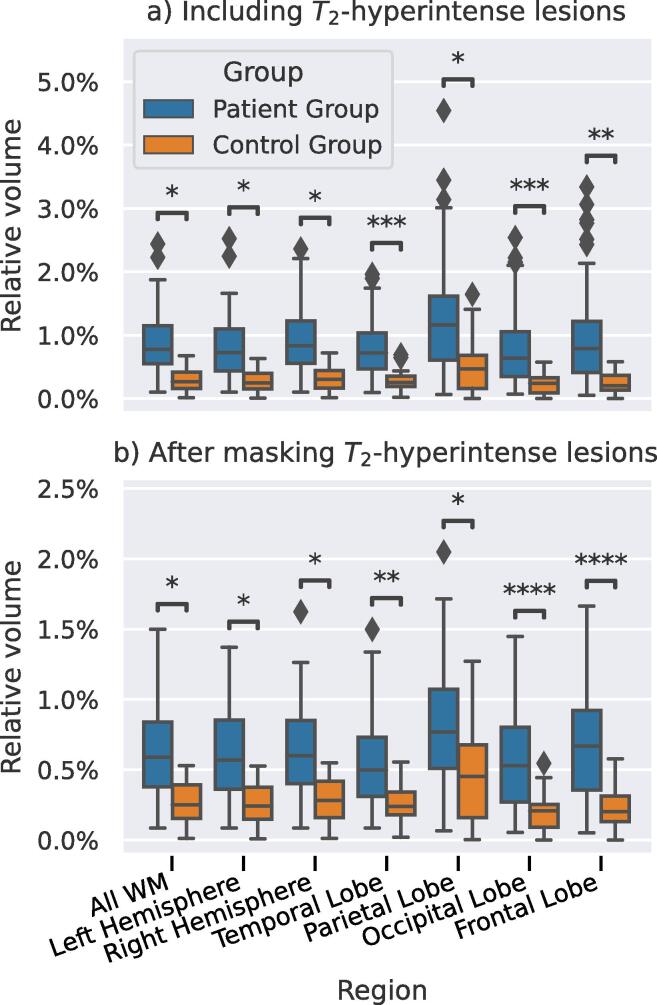
Boxplot showing the estimated relative volumes of the long- MRF-component in MS patients (blue) and controls (orange). The boxes show the quartiles of the data, the whiskers extend to the rest of the distribution, except for outliers marked with diamonds. Statistically significant difference are marked as follows: *: 0.01<p≤0.05; **: 0.001<p≤0.01; ***: 0.0001<p≤0.001; ****: p<=0.0001. (For interpretation of the references to color in this figure legend, the reader is referred to the web version of this article.)

Fig. 6a collates the estimated relative volumes of the MRF-component for the patients with MS and controls. In Table 2 the mean and standard deviations and the results of the performed generalized linear regression analyses are shown. Patients with MS had a significantly higher volume of the long component compared to controls (b = 0.0044, 95% confidence interval [0.00066 0.0081] (p = 0.02)). A similar difference was observed comparing the two hemispheres separately. We also found a higher volume of the long component in MS patients compared to controls in all individual white matter regions. The largest difference (b = 0.0052 [0.0019 0.0085], p = 0.002) was found in the frontal lobe and the smallest difference in the temporal lobe (b = 0.0039 [0.0018 0.006], p < 0.001).

**Table 2:** Generalized linear regression analyses were applied to test for differences in relative volume of the long-$T_{2}^{*}$ component between the MS patients and controls. Tests were performed for different brain regions and corrected for age and sex. A positive coefficient indicates a larger volume in the patient group. CI: confidence interval.

Brain Region	Volume in controls	Volume in patients	Regression - B-coefficient	95% CI	p-value
All	0.30%*±*0.20%	0.88%*±*0.53%	0.0044	[0.00066 0.0081]	0.022
Left Hemisphere	0.29%*±*0.19%	0.85%*±*0.52%	0.0043	[0.00059 0.0079]	0.024
Right Hemisphere	0.32%*±*0.21%	0.92%*±*0.56%	0.0045	[0.00065 0.0084]	0.023
Temporal Lobe	0.27%*±*0.17%	0.79%*±*0.44%	0.0039	[0.0018 0.006]	<0.001
Parietal Lobe	0.50%*±*0.42%	1.20%*±*0.79%	0.0048	[0.00092 0.0086]	0.016
Occipital Lobe	0.23%*±*0.15%	0.76%*±*0.53%	0.0049	[0.0023 0.0074]	<0.001
Frontal Lobe	0.25%*±*0.17%	0.92%*±*0.68%	0.0052	[0.0019 0.0085]	0.0023

Additionally, Fig. 6b and Table 3 show that this difference between the MS patients and healthy controls was also found when *T*_2_-hyperintense lesions were masked and only more subtle white matter changes were considered (b = 0.003, [0.00068 0.0053], p = 0.012). A sensitivity analysis only including patients and controls for for Site A as shown in Table S1 showed comparable results to the main analysis (p < 0.001). A second sensitivity analysis with an age-matched subset of patients (16 patients, 8 from site A, 8 from site B) was performed to test whether the between-group differences were age driven. As shown in Table S2, comparable b-values were found, but with increased p-values.

**Table 3:** Results from the generalized linear regression analyses to test for differences in relative volume of the long-$T_{2}^{*}$ component between the patients with MS and controls. Compared to Table 2 T2-hyperintense lesions were masked after which the volumes were calculated. Comparisons were made for different regions and all were corrected for age and sex. A positive B coefficient indicates a larger volume in the patient group. CI: confidence interval.

Brain Region	Volume in controls	Volume in patients	Regression B-Coefficient	95 % CI	p-value
All	0.28%*±*0.17%	0.63%*±*0.33%	0.003	[0.00068 0.0053]	0.012
Left Hemisphere	0.26%*±*0.17%	0.62%*±*0.32%	0.003	[0.00071 0.0053]	0.011
Right Hemisphere	0.29%*±*0.18%	0.65%*±*0.33%	0.003	[0.00062 0.0053]	0.014
Temporal Lobe	0.25%*±*0.14%	0.53%*±*0.28%	0.0023	[0.00095 0.0037]	0.0011
Parietal Lobe	0.46%*±*0.34%	0.81%*±*0.41%	0.0026	[0.00055 0.0047]	0.014
Occipital Lobe	0.21%*±*0.14%	0.56%*±*0.32%	0.0035	[0.0019 0.005]	<0.001
Frontal Lobe	0.23%*±*0.15%	0.68%*±*0.39%	0.0039	[0.002 0.0058]	<0.001

### 3.4 Relationship between MC-MRF and structural white matter changes on FLAIR MRI scans in patients with MS

**Fig. 7:**
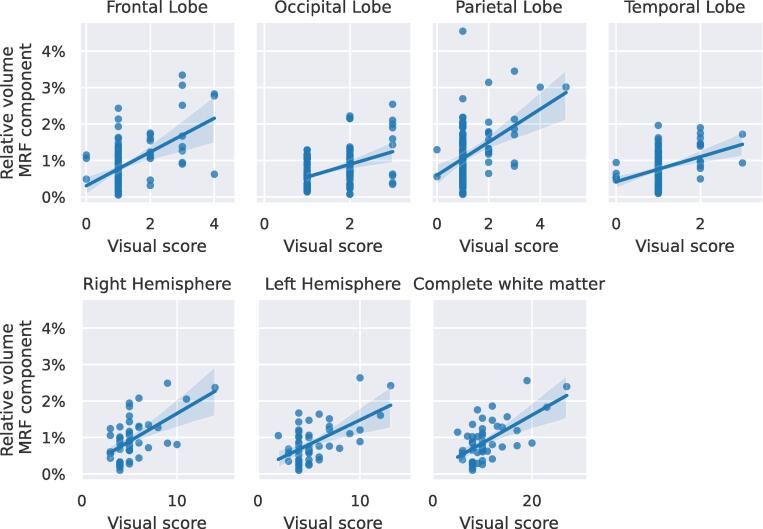
Scatter plots with linear regression estimates with 95% confidence intervals for the visual scoring of white matter damage (*x*-axis) against the volume of the long $T_{2}^{*}$-MRF-component (*y*-axis). Different plots represent different anatomical regions.

Table 4 shows the results of the Spearman correlation of the expert neuroradiologist score for amount of white matter changes and volume of the long-$T_{2}^{*}$ MRF-component, with the two sites as a confounder. Fig. 7 show scatter plots of the performed scoring with linear regression estimates for the different regions. For the total white matter volume (applying summed scores) the Spearman correlation coefficient was 0.42 (95% confidence interval: [0.16 0.63], p < 0.001). All other tested correlations showed a similar correlation in the range of 0.21–0.48 (p < 0.001). The correlation was highest in the frontal lobes and lowest in the occipital lobes.

**Table 4:** Spearman correlation analyses of the structural white matter changes scored by a neuroradiologist against the long-MRF-component volume in the white matter corrected for site. CI: confidence interval.

Region	Spearman correlation coefficient	95% CI	p-value
All	0.42	[0.16 0.63]	<0.001
Left Hemisphere	0.38	[0.11 0.6 ]	0.008
Right Hemisphere	0.40	[0.13 0.61]	<0.001
Temporal Lobe	0.33	[0.14 0.5 ]	<0.001
Parietal Lobe	0.39	[0.2 0.54]	<0.001
Occipital Lobe	0.21	[0.01 0.39]	0.04
Frontal Lobe	0.48	[0.31 0.62]	<0.001

When the volume of the MRF-component outside the *T*_2_-hyperintense lesions was compared to the visual scoring lower correlations were found in the parietal (r = 0.20 [0.01 0.39] p = 0.04) and frontal lobe (r = 0.23 [0.04 0.41] p = 0.02) as shown in Table S3.

**Fig. 8:**
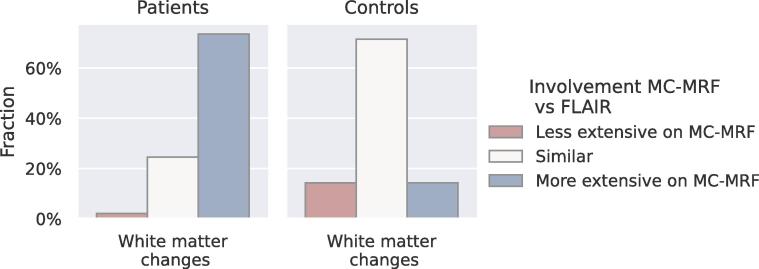
Histogram showing the visual similarity scores of the FLAIR images in comparison to the long-$T_{2}^{*}$ MRF-component map.

Fig. 8 shows the results of the performed similarity scoring of the FLAIR images and the long $T_{2}^{*}$ component. For the control group almost all $T_{2}^{*}$ component maps were scored as similar to the extent of structural white matter changes. For the patient group we observed a higher extent of the affected white matter tissue in 73% of the MRF scans compared to the structural white matter changes on the FLAIR scans. Furthermore, the estimated volumes from MC-MRF and LPA-LST differed significantly both with and without $T_{2}$-hyperintense areas ($p<10^{-16}$) and when only considering the MC-MRF component with a signal above 15% (p<0.001).

A second part of this scoring focused on *T*_2_-hyperintense regions. The similarity between regions with high fractions of the (>15%) long $T_{2}^{*}$ component fractions and the *T*_2_-hyperintense regions in the FLAIR images was high, as shown in Figure S1. A 100% similarity in volume was observed for the healthy controls and 71% for the patient data.

In secondary analyses, linear regressions were performed to test for the relationship between the volume of the MRF component and EDSS, disease duration, age and sex. Supplementary Table S4 shows the obtained results for these analyses with and without $T_{2}$ hyperintense lesions. The volume of the MRF component was associated with a higher EDSS (b = 0.00117, confidence interval [0.00027 0.0021] p = 0.012) when $T_{2}$ hyperintense lesions were included. The MRF-volume including $T_{2}$ hyperintense lesions showed a trend towards significance with disease duration (b = 0.000196, confidence interval [-0.000001 0.00039], p = 0.051).

## 4 Discussion

We performed a multi-component analysis on MRF-EPI data of MS patients and healthy controls and developed a suitable analysis pipeline to study white matter changes. In patients with MS we observed a higher volume of components with longer transverse relaxation times compared to the controls. This component correlated with structural white matter abnormalities as visible on *T*_2_-FLAIR-weighted scans in MS patients, but also showed an increased involvement of the white matter compared to the FLAIR scans. The strongest correlations were observed in the frontal lobes.

Previous multi-component models have been used in MS patients based on multi-echo spin-echo sequences sensitive to *T*_2_ effects (Laule et al., 2007a, Laule et al., 2007b). In a previous study 20 MS patients were scanned with a 48 echo multi-echo spin-echo sequence with a maximum echo time of 1*.*12 s. 27 out of 107 (25%) of the *T*_2_-hyperintense lesions showed long-*T*_2_ signal between 200 and 800 ms in 10 out of 20 (50%) of the MS patients. The total normal appearing white matter in MS patients yielded an average long-*T*_2_ signal fraction of 4*.*2% (Laule et al., 2007b). It was hypothesized in these studies that this long-*T*_2_ component reflected an increase in extracellular water, similar to the microstructural damage as observed in diffusion methods, potentially related to an increase in edema. The sequence used in these studies had extremely long echo times (around one second), resulting in acquisition times of more than 6 min per slice (without averaging).

In our study, for the first time, multi-component MRF was applied in MRF-EPI brain scans of MS patients. As described before the obtained relaxation times of white matter and gray matter were similar as observed in previous studies (Nunez-Gonzalez et al., 2022). Compared to previous results with a gradient spoiled SSFP sequence (sensitive to $T_{1}$ and $T_{2})$ a second gray matter component was estimated with short $T_{2}^{*}$, most prominently in iron-rich areas. Multiple cerebrospinal fluid components were estimated with both short and long transverse relaxation times. This split was observed before (Nagtegaal et al., 2020, Nunez-Gonzalez et al., 2022). In the ventricles this could be caused by the presence of the choroid plexus or by flow effects. In the outer regions of the brain this could potentially reflect the size of the space occupied by the fluid. The estimation of multiple myelin water like components was not expected and not observed before to our knowledge. We expect that this is partly caused by differences in inversion times per slice, but also by differences in iron content of different brain regions. In our study, we were most interested in a component with $T_{1}$ relaxation times that related to white and gray matter, and that showed increased $T_{2}^{*}$ values. In gray matter this component was also estimated, possibly reflecting that there could be similar microstructural changes in the gray matter as in the white matter. However, in the gray matter the vicinity of cerebrospinal fluid including partial volume effects might also affect the estimation. During further analysis we therefore only focused on the white matter changes in our study. With this component, we were able to visualize and quantify the extent of the microstructural white matter changes and showed that its extend is larger than the structural white matter abnormalities visible on FLAIR scans. These findings are in line with the previously stated hypothesis that in MS patients more brain tissue is affected than visible on structural MRI scans (Filippi et al., 2019, Laule et al., 2007a, Moll et al., 2011, Seewann et al., 2009). The presence around and gradual decrease from the ventricles outward of this component is in line with literature (Vaneckova et al., 2022). Multi-component relaxometry and specifically the performed MC-MRF analysis in our study has the potential to identify water-like components (i.e. with long transverse relaxation times) that are present in the microstructure of the brain. These longer relaxation times could be caused by microstructural damage resulting in an increase in extra-cellular space reducing the interaction between hydrogen protons in these spaces. This increase in extra-cellular space is potentially due to the decrease in myelin density and axonal loss (Seewann et al. 2009). The increase in the long-*T*_2_ component can also be caused by a form of edema, as was previously reported in patients with brain metastases based on Bayesian partial volume MC-MRF (Deshmane et al., 2019, McGivney et al., 2018). In a patient with brain metastases, components with *T*_2_ *>* 100 ms in peritumoral edema were measured (Deshmane et al., 2019, McGivney et al., 2018). This component showed a similar increased *T*_2_ relaxation time, indicating that at least parts of these areas might contain edema.

Earlier studies showed a significant correlation between the volume of $T_{2}$ hyperintense lesions and EDSS (Brex et al., 2002, Fisniku et al., 2008, Tourbah et al., 2001). In our study we also obtained a significant association between the long $T_{2}^{*}$ component and EDSS. However, this was only the case when the $T_{2}$ hyperintense lesions were included. An explanation for this finding could be that our study mainly consisted of patients with relapsing remitting MS and only few patients with progressive MS. This is relevant, as previous studies have shown that changes in the normal appearing white matter are more pronounced in patients with progressive MS compared to relapsing remitting MS (Absinta et al., 2015, Preziosa et al., 2014). An alternative explanation could be a power issue due to our limited group size. On the other hand the explanation could also be that the relationship is mostly driven by changes within $T_{2}$ hyperintense lesions, and less by changes within the normal appearing white matter.

Opposed to previously proposed methods, the used MRF-EPI sequence in our study allows for full brain coverage in relatively short acquisition times of less than 6 min. The MRF framework allows for many readouts with relatively short time between readouts, limiting the total scan time. Using the SPIJN algorithm multi-component estimates are obtained, where the regularization imposed by the algorithm allows for easy selection based on relaxation times. The obtained magnetization fraction maps selected based on their relaxation times from the MC-MRF method are easily interpretable due to the natural range between 0 and 1, where other parameters such as relaxation times or diffusivity require the choice of specific thresholds. The use of a fast multi-parametric method (which MRF facilitates) potentially allows for reduced variation in measurements between different sites, due to the quantitative nature and correction for inhomogeneities.

Finally, our study has several limitations. The used MRF-EPI sequence allows for an efficient k-space coverage, but the employed gradient echoes are inherently sensitive to iron and susceptibility effects (see Fig. 2 component 5). This could be a partial explanation for the differences we observe between brain regions. Future studies employing *T*_1_*, T*_2_-sensitive MRF sequences could be highly relevant in order to avoid this issue.

Also, our MRF sequence used flip angle train timings that slightly vary per slice, which may induce variation in estimated tissue fractions. However, we observed that this mainly affected the myelin water like components. A MP-PCA denoising step was used in the proposed pipeline to reduce noise in the MRF-EPI images and obtained MC-MRF maps. In preparatory experiments we observed the MC-MRF with longer $T_{2}^{*}$ relaxation times was also estimated without denoising (results not shown). This indicates that the MP-PCA denoising did not affect the obtained components, only the noise in the component maps. Another limitation could be the exact design of the used sequence, which was designed for shorter $T_{2}^{*}$ relaxation times than here reported. As a result, we observed a reduced variation in signal shape for these long relaxation times. In brief, we observed that the signal shapes of$T_{2}^{*}$ = 150 ms and $T_{2}^{*}=1$ s for $T_{1}=1$ s vary less than 1% (see Figure S2). Therefore the exact $T_{2}^{*}$ estimates as obtained have a relatively large error margin compared to the values for shorter $T_{2}^{*}$ as in for example gray and white matter. However, this did not show in the estimations of the long $T_{2}^{*}$ magnetization fractions, which we mainly studied, since the signal shapes are very similar for components with these relaxation times. Technical improvements could therefore consist of tuning the sequence to improve sensitivity for relatively long $T_{2}^{*}$ relaxation times. Another limitation of our study could be the use of visual scoring for DAWM, which we performed due to the lack of an accurate automatic quantitative method. To negate the effect of inter-observer bias the visual scorings were performed by a single, very experienced rater. Next to that, the MRI scans were performed at two different sites with slightly different acquisition settings, due to differences in available hardware. However, our sensitivity analysis showed that our main results were consistent when performed for a single site. Our study was performed in a relatively small group of MS patients. Our methods and results should therefore be validated in larger groups of MS patients with variations in disease burden, which would also allow further study of our marker in relation to disease related clinical variables.

## 5 Conclusion

Our MRF acquisition and analysis identified more white matter tissue changes in MS patients compared to controls. These tissue changes were more extensive compared to visually detectable white matter changes on FLAIR scans. The proposed method provides a better way to quantify the extent of white matter changes in MS patients, which is underestimated using only conventional clinical MRI scans. The used MRF acquisition allows for quantitative estimates in a relatively short acquisition.

## Declaration of Competing Interest

The authors declare that they have no known competing financial interests or personal relationships that could have appeared to influence the work reported in this paper.

## Data Availability

Data will be made available on request.
