# Supplementary material for: White matter changes measured by multi-component MR Fingerprinting in multiple sclerosis
Source: Neuroimage Clin. 2023 Oct 11;40:103528. doi: 10.1016/j.nicl.2023.103528 (PMC10589890; doi:10.1016/j.nicl.2023.103528)
Supplement: Supplementary data 1 [file mmc1.pdf]

6 Supporting information

Table S1. Linear regression analyses were applied to test for differences in relative volume of the long-T_2^* component between the MS patients and controls for the complete white matter (no sub-regions). In this sensitivity analysis only participants from Site A were included. A positive B coefficient indicates a larger volume in the patient group. CI: confidence interval.

Volume in controls	Volume in patients	Component	Regression B coefficient	CI[2.5%]	CI[97.5%]	p-value
0.28% ± 0.17%	0.34% ± 0.17%	T_2-hyperintensities masked	0.00336	0.00219	0.00453	<0.001
0.30% ± 0.20%	0.48% ± 0.33%	Including T_2 hyperintensities	0.00484	0.00319	0.00648	<0.001

Table S2: Sensitivity analyses consisting of linear regression analyses that were applied to test for differences in relative volume of the long-T_2^* component between the MS patients and controls in an age-matched subset of patients. Tests were performed for different brain regions and corrected for age and sex. A positive B coefficient indicates a larger volume in the patient group. CI: confidence interval.

	Brain Region	Volume in controls	Volume in patients	Regression B coefficient	95% CI	p-value
**Excluding T2-hyperintensities**	All	0.28% ± 0.17%	0.51% ± 0.32%	0.0025	[0.00024 0.0048]	0.032
	Left Hemisphere	0.26% ± 0.17%	0.49% ± 0.31%	0.0025	[0.00033 0.0047]	0.026
	Right Hemisphere	0.29% ± 0.18%	0.53% ± 0.34%	0.0025	[0.00013 0.0049]	0.040
	Temporal Lobe	0.25% ± 0.14%	0.40% ± 0.25%	0.0016	[0.00042 0.0028]	0.009
	Parietal Lobe	0.46% ± 0.34%	0.65% ± 0.39%	0.002	[-3.3^e-05 0.0041]	0.054
	Occipital Lobe	0.21% ± 0.14%	0.46% ± 0.30%	0.003	[0.0017 0.0043]	<0.001
	Frontal Lobe	0.23% ± 0.15%	0.57% ± 0.41%	0.0035	[0.0017 0.0054]	<0.001
**Including T2-hyperintensities**	All	0.30% ± 0.20%	0.69% ± 0.49%	0.0038	[0.00041 0.0071]	0.030
	Left Hemisphere	0.29% ± 0.19%	0.64% ± 0.46%	0.0036	[0.00052 0.0067]	0.024
	Right Hemisphere	0.32% ± 0.21%	0.73% ± 0.54%	0.0039	[0.00022 0.0076]	0.039
	Temporal Lobe	0.27% ± 0.17%	0.58% ± 0.38%	0.003	[0.0012 0.0048]	0.001
	Parietal Lobe	0.50% ± 0.42%	0.98% ± 0.89%	0.0041	[-1.6^e-05 0.0082]	0.051
	Occipital Lobe	0.23% ± 0.15%	0.56% ± 0.38%	0.0038	[0.0021 0.0054]	<0.001

	Frontal Lobe	0.25% ± 0.17%	0.73% ± 0.62%	0.0048	[0.002 0.0075]	0.001

Table S3: Spearman correlation analyses between structural white matter change volume score (excluding T_2-hyperintense lesions) by a neuroradiologist and estimated longT_2^*-MRF-component volume in the white matter (without T_2-hyperintense lesions). CI: confidence interval.

Region	Spearman correlation coefficient	95% CI	p-value
Left Hemisphere	0.17	[-0.12 0.43]	0.24
Right Hemisphere	0.19	[-0.1 0.45]	0.19
Temporal Lobe	0.06	[-0.14 0.26]	0.53
Parietal Lobe	0.20	[0.01 0.39]	0.04
Occipital Lobe	0.14	[-0.06 0.33]	0.16
Frontal Lobe	0.23	[0.04 0.41]	0.02

Table S4: Results of linear regression analyses between the estimated long T_2^*-MRF-component volume in the white matter (with and without white matter hyperintensities) and EDSS, disease duration, sex and age.

**Component**	**Variable**	**Regression B coefficient**	**95% CI**	**p-value**
WMHs masked	EDSS	0.000473	[-0.0001 0.001]	0.104
With WMHs	EDSS	0.00117	[0.00027 0.0021]	0.0118
WMHs masked	Disease duration	0.0000757	[-5.2e-05 0.0002]	0.238
With WMHs	Disease duration	0.000196	[-1e-06 0.00039]	0.0511
WMHs masked	Age	0.0000526	[-2.2e-05 0.00013]	0.163
With WMHs	Age	0.0000813	[-4.7e-05 0.00021]	0.21
WMHs masked	Sex	0.000198	[-0.0017 0.0021]	0.837
With WMHs	Sex	-0.0017	[-0.005 0.0016]	0.302

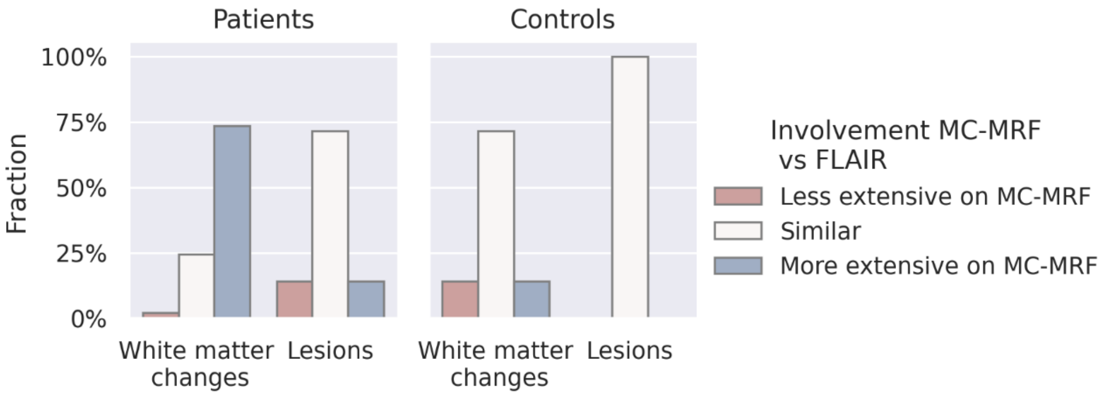

Figure S1: Histogram showing the visual similarity scores of the FLAIR scans compared to the longT_2^*MRF-component map for white matter damage and T_2-hyperintense lesions.

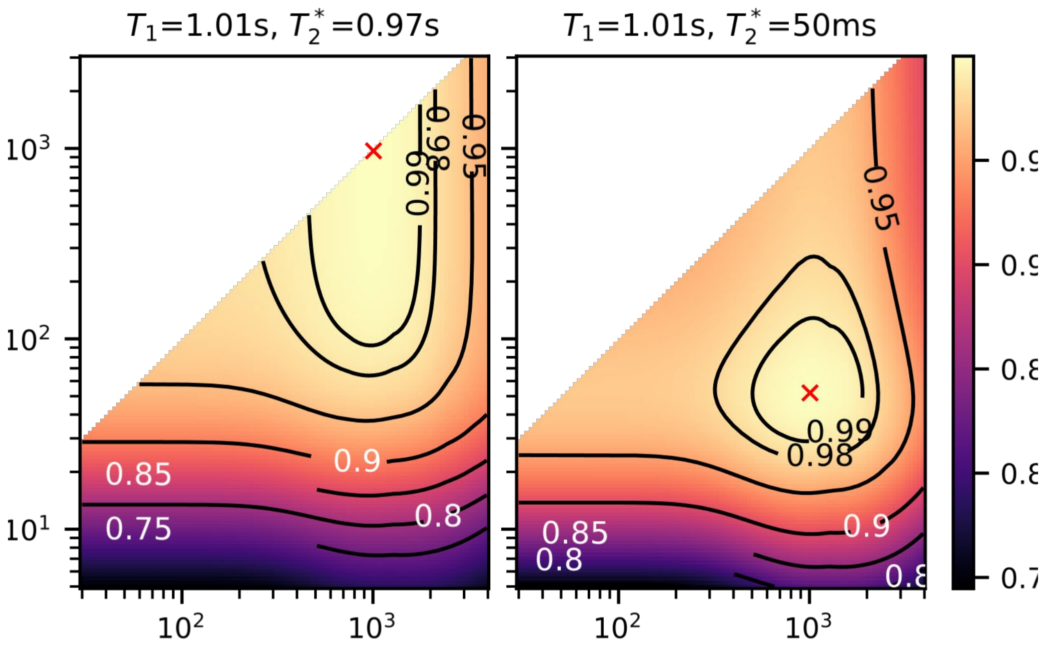

Figure S2: Similarity of two selected dictionary atom (red cross) and other atoms by computing the inner product of the normalized atoms. On the left $T_1 \approx T_2^* \approx$1s was used and on the right $T_1 \approx$ 1s and T_2^*= 50ms. A large inner product indicates that the two compared signals are very similar and as such hard to distinguish from each other. Note that a log-scaling was used for the axes. One may observe that the used MRF sequence appears to have rather low discriminative properties for longer T_2^* values. However, since the shape of the signal barely changes for small differences in estimated transversal relaxation times, this does not propagate into errors in the estimated magnetization fractions for the long T_2^* component, i.e. the sum of all dictionary atoms with the prescribed relaxation times. Effectively the exact choice of atoms and relaxation times is relatively small in this range.
